# Analysis of suspensions and recoveries of official foot and mouth disease free status of WOAH Members between 1996 and 2020

**DOI:** 10.3389/fvets.2022.1013768

**Published:** 2022-10-28

**Authors:** Aurelio H. Cabezas, Neo J. Mapitse, Paolo Tizzani, Manuel J. Sanchez-Vazquez, Matthew Stone, Min-Kyung Park

**Affiliations:** ^1^Status Department, World Organization for Animal Health, Paris, France; ^2^World Animal Health Information and Analysis Department, World Organization for Animal Health, Paris, France; ^3^Pan American Center for Foot-and-Mouth Disease and Veterinary Public Health, Communicable Diseases and Environmental Determinants of Health, Pan American Health Organization/World Health Organization, Duque de Caxias, Rio de Janeiro, Brazil; ^4^International Standards and Science, World Organization for Animal Health, Paris, France

**Keywords:** foot and mouth disease, official FMD-free status, suspension of FMD-free status, recovery of FMD-free status, WOAH Members, survival analysis

## Abstract

Foot and mouth disease was the first disease for which, in 1996, the World Organisation for Animal Health (WOAH; founded as OIE) established an official list of disease-free territories, which has helped to facilitate the trade of animals and animal products from those territories. Since that year, there have been a number of suspensions of FMD-free status which have impacted the livestock industry of the territories affected. The objective of this study is to identify factors associated with the time taken to recover FMD-free status after suspension. Historical applications submitted (between 1996 and the first semester of 2020) by WOAH Members for recognition and recovery of FMD-free status were used as the main source of data. Only FMD-free status suspensions caused by outbreaks were considered. Data on the Member's socio-economic characteristics, livestock production systems, FMD outbreak characteristics, and control strategies were targeted for the analysis. The period of time taken to recover FMD-free status was estimated using Kaplan–Meier survival curves. A Cox proportional hazard model was used to identify factors associated with the time taken to recover FMD-free status after suspension. A total of 163 territories were granted official FMD-free status during the study period. The study sample consisted of 45 FMD-free status suspensions. Africa and the Americas accounted for over 50% of FMD-free status suspensions, while over 70% of these occurred in formerly FMD-free territories where vaccination was not practiced. The study noted that implementing a stamping-out or vaccination and remove policy shortened the time to recover FMD-free status, compared with a vaccination and retain policy. Other variables associated with the outcome were the income level of the Member, Veterinary Service capacity, time taken to implement control measures, time taken until the disposal of the last FMD case, whether the territory bordered FMD-infected territories, and time elapsed since FMD freedom. This analysis will contribute toward the understanding of the main determinants affecting the time to recover the FMD free status of WOAH Members and policy processes for FMD control and elimination.

## Introduction

The World Organisation for Animal Health (WOAH) is the intergovernmental organization responsible for improving animal health, veterinary public health and animal welfare throughout the world (1, 2). WOAH is recognized by the World Trade Organization (WTO) as the global authority for defining sanitary rules in relation to animal health and zoonoses to facilitate the safe international trade of animals and animal products while avoiding unnecessary impediments to trade (3, 4). Among its other mandates, and since 1994, WOAH officially recognizes countries and zones^1^ as being free from disease for the purposes of international trade.

Foot and mouth disease is a highly infectious disease that affects cloven-hoofed animals, and it is considered one of the most devastating diseases for livestock as the virus spreads easily among susceptible populations. Beyond its implications for animal health, FMD threatens national economies and the economic livelihoods of millions of people who depend on livestock for their income (5). In 1996, FMD became the first disease in WOAH's official list of disease-free countries and zones, based on a transparent, science-based and impartial procedure for the recognition and maintenance of FMD-free status. The voluntary procedure for official recognition of FMD-free status allows WOAH Members (Members) to apply for two categories of FMD-free status for their country or a zone within their country: FMD-free status where vaccination is not practiced and FMD-free status where vaccination is practiced. Members requesting official recognition of their FMD-free country or zone status must submit an application that follows the Standard Operating Procedures established by WOAH and provide documented evidence demonstrating compliance with the Terrestrial Animal Health Code (Terrestrial Code). The FMD-free status granted by WOAH represents a milestone in the economy of Members as it facilitates the trade of animals and animal products from those territories to attractive markets that require FMD-free status (1).

Foot and mouth disease outbreaks in FMD-free recognized countries or zones would result in suspension of that territory's FMD-free status. This loss of the status results in an immediate loss of export markets that require FMD-free status, which can only be recovered once the status is restored. Moreover, the process for regaining FMD-free status could involve significant investment and activity by the Member. A number of studies have estimated the costs associated with FMD outbreaks in non-endemic countries (5–14). These costs are incurred at the production level as stamping-out polices are often implemented to combat the disease, and through disease eradication efforts and losses in revenue because of trade restrictions (8). The FMD-free status of the country or zone can be recovered by submission of an application by the Chief Veterinary officer to WOAH providing sufficient evidence that the country or zone complies with the provisions in the Terrestrial Code. In short, it is necessary to present sufficient evidence to demonstrate the absence of FMD in that country or zone and to show that there are appropriate measures in place to avoid introduction.

Foot and mouth disease has been widely distributed around the world, as discussed by Grubman and Baxt (15), Paton et al. (16), and Brito et al. (17). While FMD mostly affects countries to which the disease is endemic, countries with an FMD-free status have also been impacted by the incursion of the virus. The FMD outbreaks in Chinese Taipei (6, 18); South Korea (19–22); Japan (23); the United Kingdom (UK) (24–26); France (27); Ireland (28); the Netherlands (29); South Africa (30); Uruguay (31); and Argentina (32) represent a few examples in which an FMD outbreak has led to the suspension of officially recognized FMD-free status. However, despite the research conducted to describe and understand the epidemiology of these outbreaks, the circumstances that led to the suspension and the strategies used for the subsequent reinstatement of FMD-free status have not yet been comprehensively described.

Several studies can be found in the literature that attempt to evaluate strategic approaches that could affect FMD-free status recovery periods. One study assessed the quality of higher potency vaccines and the performance of DIVA (differentiating infected from vaccinated individuals) assays on post-outbreak serosurveillance (33). Other authors explored the impact of using emergency vaccination during an epidemic in endemic and non-endemic countries (34), and the impact of emergency vaccination on the waiting period to recover FMD-free status (35). The effects of post-outbreak management strategies for vaccinated animals on market trade have also been explored (36). Studies using mathematical modeling have been conducted to simulate outbreaks in the Netherlands, with the application of a vaccination and retain policy, to evaluate the dynamics of the simulated outbreaks and to assess that policy's effect on regaining FMD-free status (37, 38). Finally, there have been studies evaluating surveillance methods to substantiate the absence of disease and viral circulation after FMD outbreaks in FMD-free territories (39–41). However, it is difficult to extrapolate conclusions from the above studies that would apply to a range of different scenarios, as they evaluated specific cases. It is also important to consider the intrinsic characteristics of a country or zone and the capability of a country to manage these emergency events when making informed recommendations on control strategy policies.

The objectives of this study are to identify factors associated with the time taken to recover a country or zone's FMD-free status after its suspension as the result of an outbreak, and to use that information to make informed recommendations on areas that should be strengthened for better preparedness and contingency planning against a potential incursion of FMDv. This is the first study that utilizes all the historical records available on the Member submissions to WOAH for FMD-free status recovery.

## Materials and methods

### Case selection

A country or zone was considered as the study unit. The source population consisted of all study units officially recognized as FMD-free (with or without vaccination) between 1996 and the first semester of 2020 (inclusive). Study units that had been granted an official FMD-free status, had their FMD-free status suspended as the result of an FMD outbreak and had applied for recovery of FMD-free status were included in the study. Study units that applied a zoning strategy—after the suspension—which resulted in the recovery of FMD-free status in only a part of the initially recognized country or zone and study units with no records available were excluded from the study.

### Data collection

The main source of data for this study were the dossiers submitted to WOAH by Members for recognition and recovery of FMD-free status during the study period. Other important sources of data were the immediate notifications and follow-up reports of exceptional epidemiological events submitted to WOAH during the study period, retrieved from two digital interfaces: Handistatus II^2^ which records data between 1996 and 2004 and WAHIS^3^ which records data between 2005 and 2020. Other sources of data were FAOSTAT,^4^ DataBank,^5^ and other relevant WOAH reports. The analysis targeted variables in three main groups: agricultural characteristics of the study units, characteristics of the FMD outbreak, and emergency response and preparedness of the study unit (see Table 1 for more detail about targeted variables). All data collected were contemporaneous with the period of suspension/recovery of FMD-free status in the study unit. Data were compiled in Microsoft Excel^®^ 365 (Microsoft, Redmond, WA, USA).

**Table 1:** Target variables for the analysis.

**Group**	**Variable**	**Scale of measurement**
Agricultural characteristics of the study unit	Epidemiological unit	Farm, village, other
	Livestock density	Number of livestock per km^2^ of agricultural land
	Shared borders with neighboring FMD-infected countries or zones	Binary
Characteristics of the FMD outbreak	FMDv serotype	A, O, C, SAT 1, SAT 2, SAT 3, or ASIA 1
	Species in which FMD was first detected	Bovines, swine, or small ruminants
	Species affected during the FMD outbreak	Bovines, swine, small ruminants, multiple
	Percentage of at-risk livestock during the outbreak	Percentage of confirmed FMD cases, and percentage of animals culled (if only stamping-out was applied) or proportion of vaccinated animals (if only emergency vaccination was applied) or percentage of animals culled and vaccinated (if stamping-out and emergency vaccination were applied) in relation to the total livestock population
Emergency response and preparedness of the study unit	Income level	Higher, upper-middle, lower-middle, low
	Time since FMD freedom^a^	Number of years
	Capacity of official Veterinary Services	Number of official veterinarians per number of livestock
	Time taken to implement control measures after FMD detection	Number of days
	Time between first detection of FMD and culling or vaccination of the last case	Number of weeks
	Time since adoption of FMD legislation or latest revision prior to suspension of FMD-free status	Number of years
	Control strategy used during the outbreak	Stamping-out, emergency vaccination and retain^b^, emergency vaccination and remove^c^
	Conduction of simulation exercises prior suspension	Binary
	Conduction of simulation modeling studies prior suspension	Binary
	Existence of a public private partnership^d^	Binary

a Refers to the time elapsed since the date of initial recognition for countries or zones that had only one suspension, or since the date of last suspension for countries or zones with more than one suspension.

b Refers to letting vaccinated animals complete their production cycle after the application of emergency vaccination to control FMD outbreaks (protective vaccination).

c Refers to the slaughter of vaccinated animals after the application of emergency vaccination to control FMD outbreaks (suppressive vaccination).

d A joint approach in which the public and private sectors agree responsibilities and share resources and risks to achieve common objectives that deliver benefits in a sustainable manner.

### Statistical analysis

Descriptive statistics were computed to explore variables gathered from the data sources. Means, medians, and percentiles were computed for continuous variables while frequency tables were computed for categorical variables.

**Figure 1:**
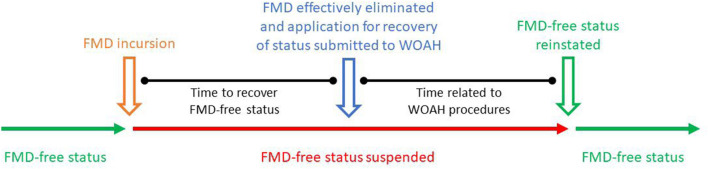
Schematic figure showing the relevant timelines in the study.

The association of potential risk factors affecting the time taken to recover FMD-free status (the outcome) was determined by conducting a survival analysis. The outcome was modeled in months and calculated from the date of the suspension of FMD-free status until the date of submission of the application for recovery of FMD-free status (see Figure 1). The date of submission of the application for the recovery of FMD-free status was used instead of the date of the official recovery to avoid administrative procedures by WOAH affecting the analysis. Thus, the date of application was taken as the moment when the country/zone were ready to fulfill the requirements for status recovery.

The 2020 edition of the FMD Chapter in the Terrestrial Code stipulates that Members can apply for the recovery of FMD-free status within 24 months after the date of suspension. If FMD-free status cannot be recovered within this period, Members would need to follow the general provisions for recognition of FMD-free status. However, this deadline of 24 months was only described in the Terrestrial Code editions of 2002 and from 2015 onwards. It was noted that the time-to-application for recovery after suspension was within 24 months in 75% of study units; within 36 months in 90% of the study units and up to 5 years for the remaining 10% of study units. Considering that this deadline was not described in the FMD Chapter of all the editions of the Terrestrial Code of the study period, a threshold of 36 months was used for the purposes of the study. Study units for which an application for recovery was not submitted within 36 months after suspension or by the end of the study period were progressively right censored.

The time taken to recover FMD-free status upon suspension according to the different factors was explored using Kaplan–Meier survival curves. In a further analysis, a Cox proportional hazard model was constructed. Variables with a large proportion (over 60%) of missing values were excluded from the analysis, and pair-wise correlations were also explored to assess for collinearity. Univariable models with each predictor and the outcome were determined to be fit to assess for unconditional associations, and associations with a liberal *p*-value ≤ 0.2 were selected for the multivariable model. Selection for retention in the model was carried out by the manual forward selection process, using a level of 0.05 as a criterion for statistical significance. Two-way interaction terms were evaluated. Evaluation of the proportional hazard assumption was conducted by estimating the Schoenfeld residuals and a test for significance for non-zero slope (log hazard–ratio function is constant over time) (42). The overall fit of the model was evaluated by computing the Groennesby and Borgan goodness-of-fit test, while the predictive ability of the model was assessed by computing the Harrell's C concordance statistic (42). Outliers and influential observations were evaluated by computing deviance and score residuals. Shared frailty models were also fitted for the assumption of non-independence between study units. “Member” was included as a frailty term to deal with the lack of independence for multiple failures within a Member—a Member having more than one suspension of FMD-free status, or more than one zone having a suspension of FMD-free status. The “edition of the Terrestrial Code” was also included as a frailty term with the assumption that study units for which applications for recovery were assessed under the requirement in a specific edition of the Terrestrial Code were likely correlated (see Supplementary Table A1 for a summary of the variation of waiting periods in the Terrestrial Code since 1996). The contribution of the frailty component to the model was evaluated by the log-likelihood test of θ = 0 (equal variances) for evidence of within-cluster correlation. If there was no statistical significance (*p*-value > 0.05), then the simpler model was preferred. Results for significant variables are presented with hazard ratios, 95% confidence intervals, *p*-values, and medians for the time between FMD-free status suspension and application for recovery of FMD-free status. Data cleaning and statistical analysis were conducted in STATA 13 (StataCorp LP, College Station, TX, USA), the output figures were done in R (43) using the ggplot package, and the map was drawn in ArcGIS 10.3.1 (Esri, Redlands, CA, USA).

## Results

### Descriptive analysis

**Figure 2:**
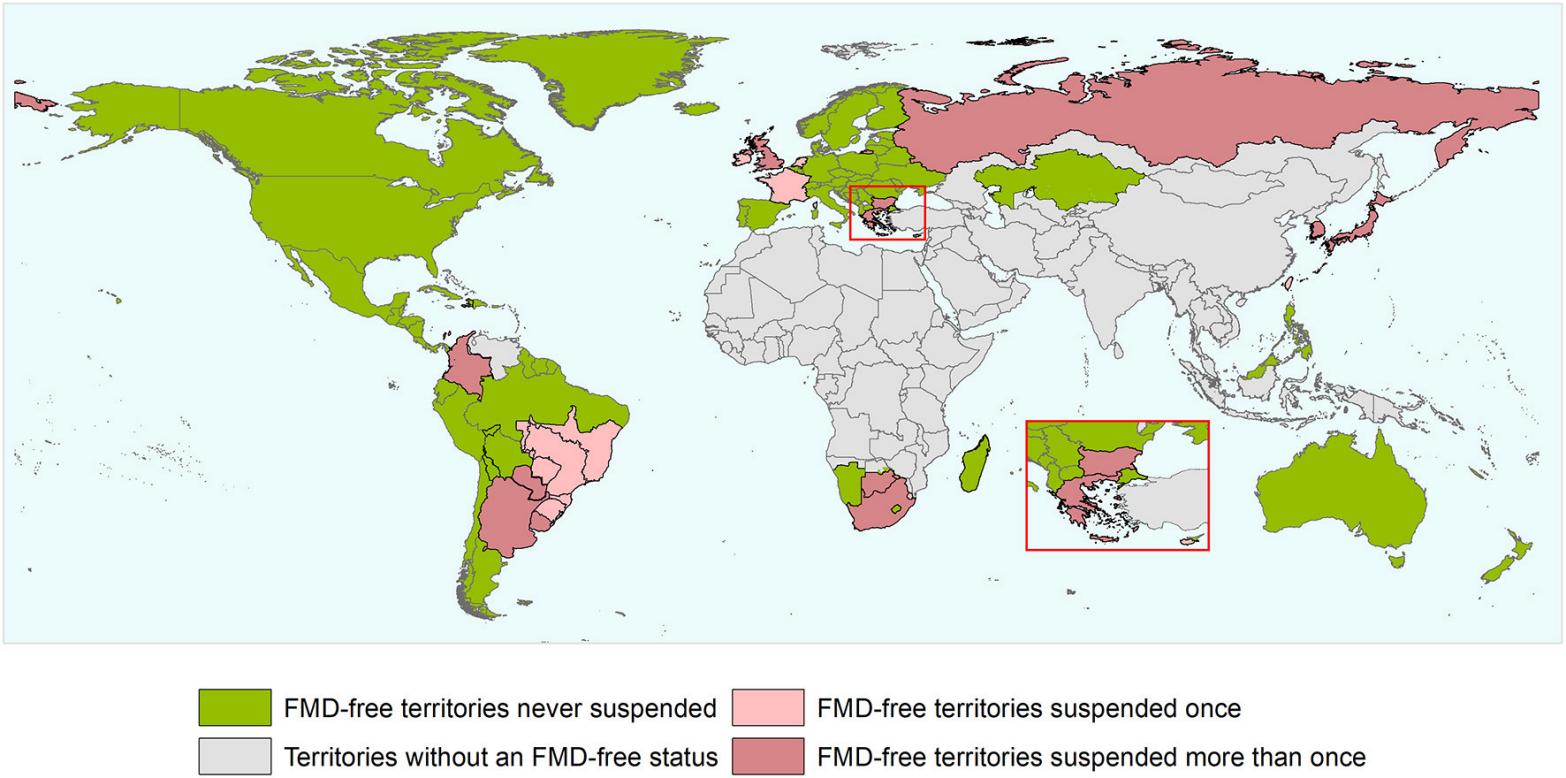
World map depicting the FMD-free status of countries and zones during the study period (1996 and the first semester of 2020). Territories suspended for reasons others than an outbreak and/or that did not meet the inclusion criterium are not depicted in the map as suspended territories. The authors highlight that the countries and zones depicted in the map does not necessarily represent the list of countries and zones having an official recognized FMD-free status by WOAH after the study period.

**Figure 3:**
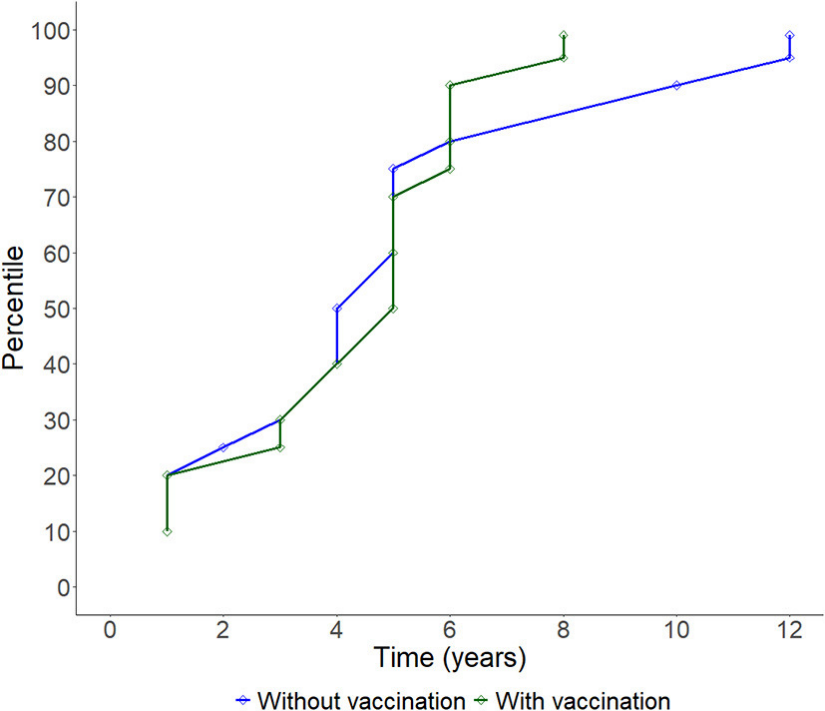
Line plot showing the cumulative progression of study units having their FMD-free status suspended over time.

**Figure 4:**
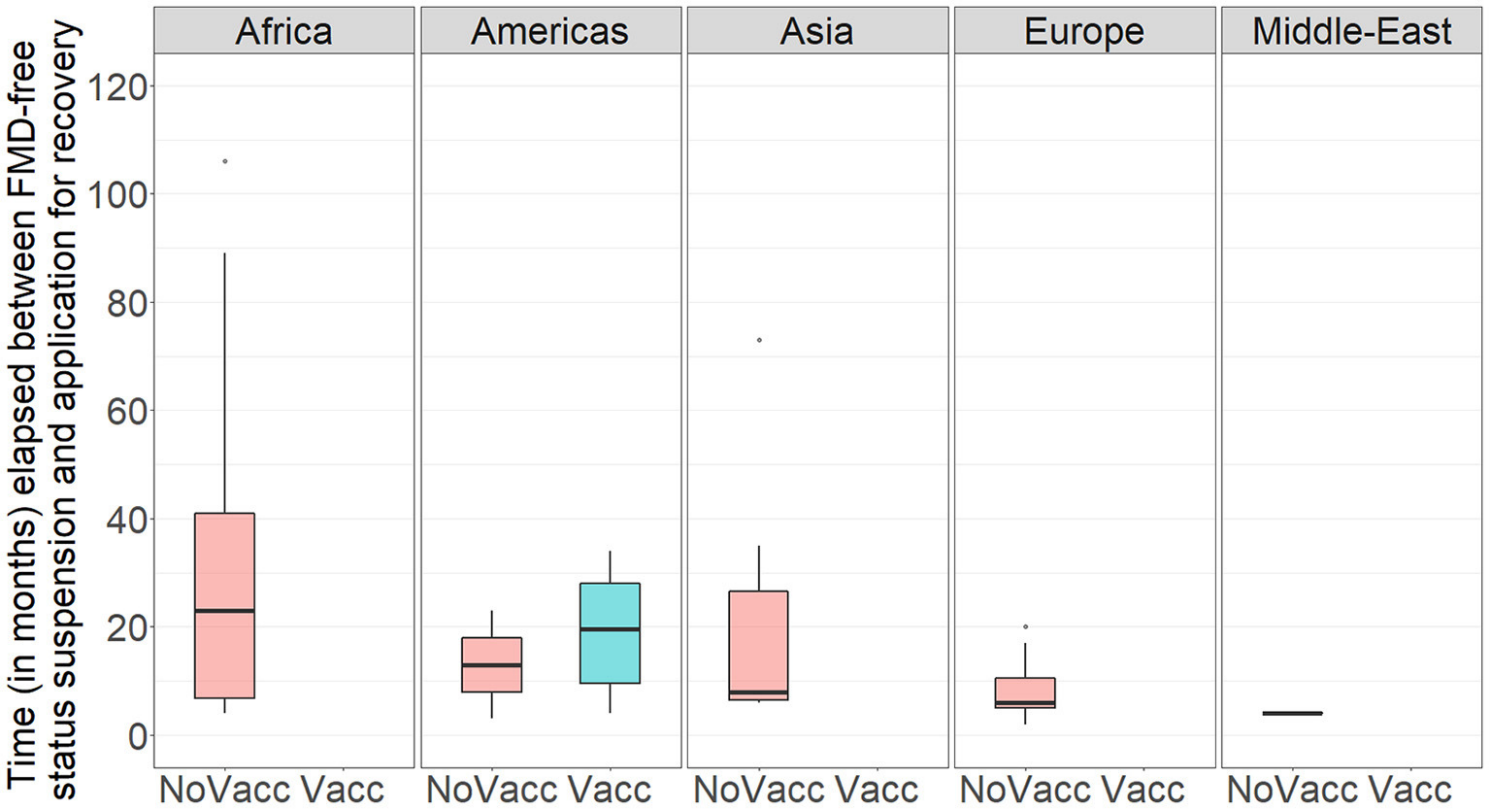
Boxplot showing the time (in months) between suspension of FMD-free status and application for recovery of FMD-free status in the study population per WOAH Regional Representation. NoVacc means FMD-free status without vaccination, Vacc means FMD-free status with vaccination.

**Figure 5:**
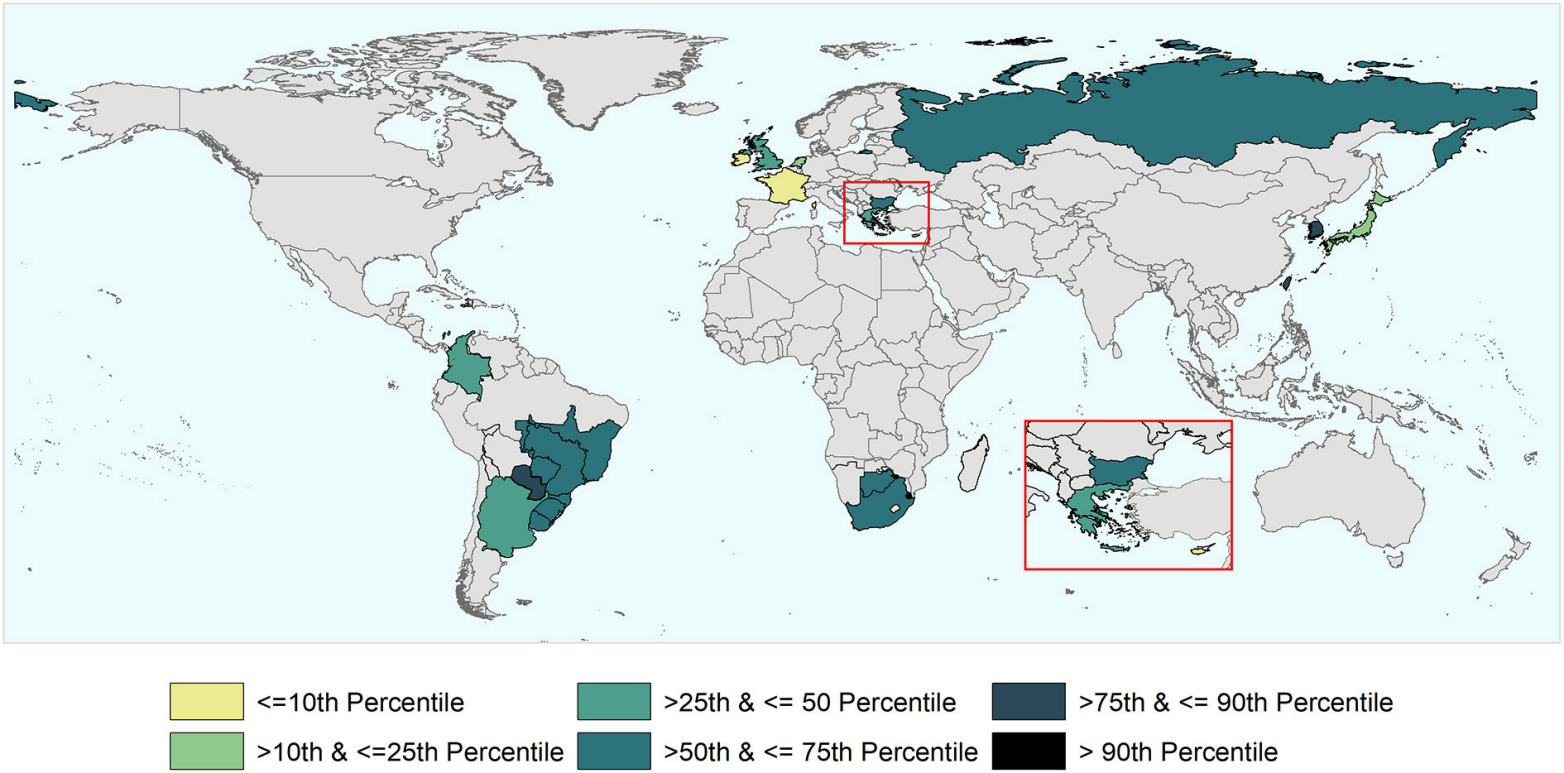
World map depicting the cumulative time taken to recover (i.e., to submit to the WOAH the official request) the FMD-free status suspended in the study units that were part of the analysis. The time to recover ranged between 3 and 106 months and percentiles were used for the categorization of study units.

During the study period, there have been 163 official FMD-free statuses granted to countries or zones (see Table 2). FMD-free countries and zones without vaccination represent 44 and 26%, respectively, while FMD-free countries and zones with vaccination represent 4 and 26%, respectively. A total of 52 suspensions of FMD-free status have taken place, of which 45 suspensions met the inclusion criteria and were therefore part of the analysis (*n* = 45 study units). Zones accounted for 51% (23) of suspensions while countries for 49% (22). Seventy-three percent (33) of suspensions took place in study units with FMD-free status without vaccination; thus, 27% (12) of suspensions were in study units that were FMD-free with vaccination (see Table 2). The number of suspensions in each study unit ranged from 1 to 4, with a median of 1 (see Figure 2). It was found that in 80% of the study units (free with and without vaccination), the status was suspended within 6 years after recognition (see Figure 3). The time from suspension of FMD-free status to application for recovery ranged from 3 to 106 months, although in 90% of study units, this time was < 36 months (see Figure 4). The cumulative time to recover the status in 90% of study units was < 72 months (see Figure 5).

**Table 2:** Number of official FMD-free status recognitions and suspensions during the study period.

**Official category of FMD-free status recognized**	**Number of official FMD-free status recognitions (%)**	**Number of suspensions and recoveries of FMD-free status (%)**
Free country without vaccination	73 (44%)	21 (47%)
Free country with vaccination	6 (4%)	1 (2%)
Free zone without vaccination	42 (26%)	12 (27%)
Free zone with vaccination	42 (26%)	11 (24%)

Most of the outbreaks that led to suspensions were caused by FMD serotype O (71%). The population reported to be at-risk during the FMD outbreak(s) was < 8% of the total population in the study units. Over 40% (18) of study units that had their FMD-free status suspended applied stamping-out alone as a strategy to control the FMD outbreak(s), while 4% (2) applied emergency vaccination only, and 56% (25) applied a combination of stamping-out and emergency vaccination. In 49% (22) of the outbreaks that led to suspensions, bovines were the only species affected while in 40% (18) FMD infected multiple species. Simulation exercises were conducted in 8% (4) of study units prior to the suspension while simulation modeling studies to explore control strategies against potential FMD outbreaks were conducted prior to suspension in 18% (8) of study units. In study units that conducted simulation exercises, these occurred 4–8 years prior suspension. A public private partnership (PPP) relevant to FMD was in place in 24% (11) of the study units. In 9% (4) of the study units, a PPP was in place but the year of start of the PPP could not be determined. A concise summary of the data collected can be found in Supplementary Tables A2, A3.

### Survival analysis

**Figure 6:**
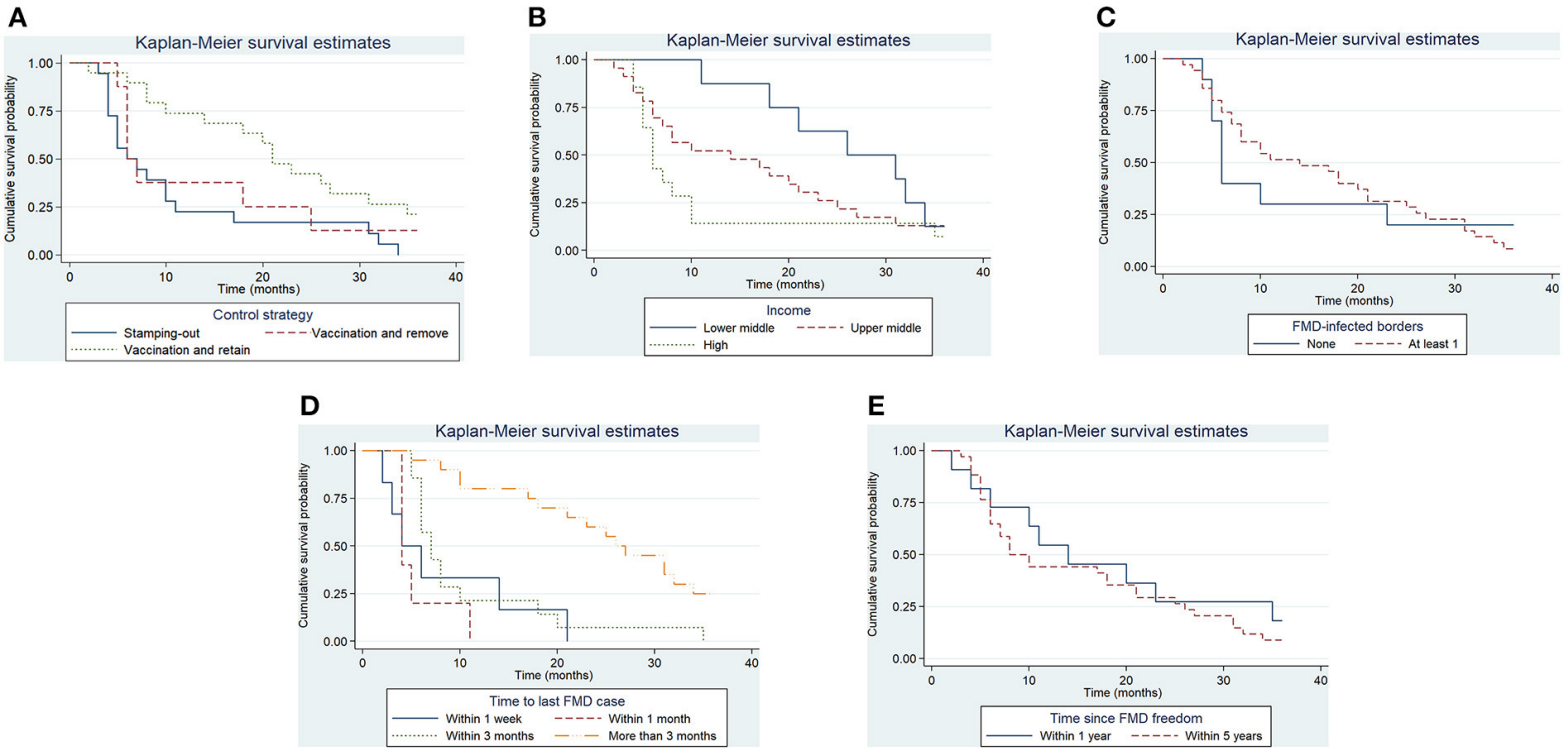
Kaplan–Meier survival function for significant categorical variables. Control strategy implemented to control the outbreak **(A)**, Income level of Member **(B)**, whether the study unit shared at least one border with an FMD-infected country or zone **(C)**, the time elapsed in the study unit between the detection of FMD and the slaughter or vaccination of the last FMD case **(D)**, the time since the study unit was last recognized as having FMD-free status **(E)**.

A total of 45 suspensions of FMD-free status were included in the analysis, from which 88% (40) recovered FMD-free status and 12% (5) of those were right censored. The total time at risk (that study units had their FMD-free status suspended until the status was recovered or until the study units were right censored) was 723 months. Study units of Members with a high-income level had a median survival time of 6 months, compared to 14 (upper-middle income) and 26 (low-middle income). The use of stamping-out (only), or stamping-out combined with emergency vaccination and remove policy, had a median survival time of 6 months, compared to 21 months in which stamping-out was implemented in combination with emergency vaccination and retain policy. Study units in which suspension of FMD-free status occurred after a year or longer than when the FMD-free status was recognized, had median survival time of 8 months compared to 14 months for those units in which suspension occurred within 1 year after FMD-free status recognition. More detail on the median and interquartile range (IQR) of survival times are displayed in Table 3. The Kaplan–Meier survival functions are presented in Figure 6. The results of the Cox proportional hazards model univariable analysis are presented in Table 4. A total of 8 variables were selected for the multivariable Cox proportional hazard model (see Table 5). The inclusion of “Member” and “Edition of the Terrestrial Code” as a frailty term in the multivariable model were not statistically significant, so a simpler model was chosen, and those results are described.

**Table 3:** Summary statistics for significant categorical variables in the univariable analysis, showing the median and interquartile range for the survival time (months) taken to recover FMD-free status.

**Variable**	**Category**	**Number of study units**	**Survival time**^**a**^ **(months)**		
			**25th percentile**	**Median**	**75th Percentile**
Income level	Lower middle	8	18	26	32
	Upper middle	23	8	14	25
	High	14	5	6	10
Time between first detection of FMD and culling or					
vaccination of the last case	Within 1 week	6	3	4	14
	Within 1 month	5	4	4	5
	Within 3 months	14	6	7	10
	More than 3 months	20	17	26	34
Control strategy used during					
the outbreak	Stamping-out	18	4	6	11
	Stamping-out + vaccination and remove	8	6	6	18
	Stamping-out + vaccination and retain	19	10	21	35
Shared borders with neighboring FMD-infected					
countries or zones	None	10	5	6	23
	At least 1	35	6	14	27
Time since FMD-freedom	1 year	11	6	14	35
	More than 1 year	34	6	8	26

a The survival time is the time in months between suspension of FMD-free status and application for recovery of FMD-free status.

**Table 4:** Results of the univariable analysis to estimate the association between targeted variables and the time taken (in months) to recover FMD-free status.

**Variable**	**Category**	**Hazard ratio**	***p*-value**	**95% CI**
Income level	Lower middle			
	Upper middle	1.67	0.244	0.70–3.98
	High	2.58	0.049	1.01–6.58
Species in which FMD was first detected	Bovines			
	Small ruminants	3.76	0.037	1.08–13.09
	Swine	0.90	0.814	0.37–2.18
	Wild	1.41	0.741	0.19–10.56
	Multiple	2.45	0.149	0.73–8.28
Time taken to cull or vaccinate the last FMD				
case after FMD detection	Within 1 week			
	Within 1 month	1.78	0.356	0.52–6.06
	Within 3 months	0.63	0.357	0.24–1.68
	More than 3 months	0.17	0.001	0.06–0.46
Control strategy used during the outbreak	Stamping-out			
	Stamping-out + vaccination and remove	0.68	0.394	0.28–1.64
	Stamping-out + vaccination and retain	0.38	0.006	0.18–0.76
Shared borders with neighboring				
FMD-infected countries or zones	At least 1			
	None	1.03	0.19	0.44–2.09
Time since FMD freedom	1 year			
	More than 1 year	1.32	0.16	0.63–2.79
Time since adoption of FMD legislation or latest revision prior to suspension of FMD-free status (years)		0.97	0.042	0.96–0.99
Capacity of official veterinary services^a^		1.01	0.021	1.01–1.03
Time taken to implement control measures after FMD detection (days)		0.95	0.122	0.88–0.01
Percentage of at-risk livestock during the outbreak		0.98	0.215	0.95–1.01

a The capacity of official Veterinary Services for the purpose of this study was estimated as the number of official veterinarians per 100,000 livestock in the study unit.

**Table 5:** Results of the multivariable analysis to estimate the association between selected variables from the univariable analysis and the time taken (in months) to recover FMD-free status.

**Variable**	**Category**	**Hazard ratio**	***p*-value**	**95% CI**
Income level	Lower middle			
	Upper middle	5.51	0.001	1.35–8.08
	High	6.08	0.022	1.59–9.04
Time taken to cull or vaccinate the last FMD				
case after FMD detection	Within 1 week			
	Within 1 month	0.92	0.928	0.16–5.34
	Within 3 months	0.33	0.164	0.07–1.57
	More than 3 months	0.08	0.001	0.04–0.12
Control strategy used during the outbreak	Stamping-out			
	Stamping-out + vaccination and remove	0.41	0.218	0.01–1.69
	Stamping-out + vaccination and retain	0.11	0.001	0.08–0.41
Shared borders with neighboring				
FMD-infected countries or zones	At least one			
	None	2.22	0.068	0.83–6.94
Time since FMD freedom	1 year			
	More than 1 year	5.8	0.011	1.51–10.63
Capacity of official veterinary services		1.05	0.03	1.02–1.08
Time taken to implement control measures after FMD detection (days)		0.89	0.015	0.83–0.98
Percentage of at-risk livestock during the outbreak		0.96	0.003	0.94–0.98

In reporting hazards of recovery resulting from this survival analysis, note that shorter survival represents faster recovery of freedom, and therefore higher hazards of recovery represent the more favorable outcome. In other words, higher hazard ratios are indicative that study units in a given category were more likely to have a faster recovery when compared to study units in the baseline category. For study units of a Member with an upper middle- or high-income level, the hazards of recovery of FMD-free status were 5.5 (95% CI, 1.5–8.08) and 6 (95% CI, 1.59–9.04) times greater than for study units of Members with a lower middle-income level, that is, study units with an upper-middle or high-income level had faster recoveries. The hazard for recovery of FMD-free status in study units that managed to slaughter or vaccinate the last FMD case within 3 months was 0.08 (95% CI, 0.04–0.12) times the hazard (a 90% decrease) when compared to the baseline (slaughter or vaccination of the last FMD case within a week). The implementation of stamping-out combined with emergency vaccination and remove policy was not significantly different from the implementation of stamping-out alone. However, the hazard for recovery for stamping-out in combination with emergency vaccination and retain policy was 0.11 (95% CI, 0.08–0.41) times the hazard (an 89% decrease) when compared to implementing stamping-out only. Study units that shared no borders with FMD-infected countries or zones had 2.2 (95% CI, 0.83–6.94) times the hazard to recover their FMD-free status, when compared to study units that shared a border with an FMD-infected country or zone. Study units in which FMD-freedom (either initial recognition or recovery) was achieved longer than a year prior to the suspension of status had 6 times the hazard (95% CI, 1.51–10.63) to recover their FMD-free status when compared to study units in which FMD-freedom was achieved within a year of the suspension. An increase of one official veterinarian in charge of the animal health situation in the country or zone per 100,000 livestock increased the hazard to recover FMD-free status by 5% (95% CI, 2–8%). Moreover, an increase of 1 day in implementing measures after FMD detection decreased the hazard to recover FMD-free status by 11% (95% CI, 2–17%). An increase in 1% of the livestock population at risk in the study unit decreased the hazard to recover FMD-free status by 4% (95% CI, 2–6%).

Interaction terms included in the multivariable model were not found to be statistically significant and were therefore removed from the final model. The statistical test to evaluate the assumption of proportional hazards suggested that there was no evidence that the assumption was violated (*p*-value 0.92). The Groennesby and Borgan goodness-of-fit test produced a *p*-value of 0.7, which suggested that there was no evidence of lack of fit. The Harrell's C concordance static was 0.89, which suggested that the model correctly predicted the findings 89% of times. There were no outliers and/or substantial influential observations identified.

## Discussion

The current study documents the suspensions, and recoveries of FMD-free status from 1996, when WOAH first started granting official FMD-free status to its Members, until the first semester of 2020. Information has been synthesized from official documentation submitted by Members to WOAH and some external sources. This unique study has allowed to explore and understand the risk factors that could affect the time taken to recover FMD-free status after it had been suspended, and to use that understanding to help national veterinary services to make informed decisions to manage FMD at the country level. This discussion starts addressing general findings at the descriptive analysis, then continues to discuss the main findings at the survival analysis, and concludes with limitations of the study.

### General findings

Our results show that 89% of the FMD-free status suspensions occurred between 1996 and 2011. After 2011, there have been only sporadic suspensions, which reflects the effort and progress made by Members in the control and prevention of FMD, potentially including better implementation of the expanded range of risk management options provided in the Terrestrial Code. For instance, in South America, significant progress has been made over recent years and continues to be made, thanks to an eradication program led by the Pan American Foot and Mouth Disease Center (PANAFTOSA), which targets improvements in veterinary infrastructure, mass vaccination campaigns, and PPPs to eliminate FMD (31, 44–46). In other regions, on the contrary, the management and control of FMD has been more challenging. For instance, in Asia the disparities across the continent in the financial resources allocated to Veterinary Services have had a direct impact on efforts to control and eliminate FMD (47). In parts of Africa and Eastern Europe, the role of seasonal transhumance (48) and wildlife species such as African buffalo (*Syncerus caffer*) and wild boar (*Sus scrofa*) in the epidemiology of FMD have also affected FMD control (49–52). Serotype O has been found to be responsible for most of the suspensions of FMD-free status (66%), which is not surprising as it is the most widely spread serotype around the world (15–17, 53).

In regard to the percentage of livestock population at risk during FMD outbreaks, it was found that fewer than 8% of the total livestock population in each study unit were considered at risk, and 75% of outbreaks were localized events, which means that they were restricted to a limited area of the study unit. Considering the relatively low percentage of livestock affected and at risk, and the localized nature of these outbreaks, it is pertinent to ask why Members did not opt to apply for the establishment of a containment zone (CZ) as a strategy to hasten the recovery of at least part of their territories. Since the inclusion of provisions for the establishment of a CZ in the 2008 edition of the Terrestrial Code, this approach has been implemented in only three cases (17%).

One notable approach that has been used in the past few years, in a range of different territories and livestock production systems, is the application of network analysis using routinely or specifically collected traceability data to understand patterns of livestock movements (54–65). These methods can be helpful to identify areas within a country that are at a higher risk of the spread of FMDv or of being infected during an FMDv incursion. This information could be useful in determining the boundaries of a CZ that could be established as a strategy to quickly recover FMD-free status in part of a Member's territory.

### Main findings

Based on the data and methods used, there was evidence of an inverse association between the income level of Members and the time taken to recover FMD-free status. This could be due to Members with a higher income level having more resources to devote to surveillance and early detection systems that lead to rapid FMD detection and the swift implementation of control measures, in addition to more resources being available for an emergency response in the event of disease outbreaks. Although the authors consider this finding plausible, this should not be over-emphasized because the study made use of an overall classification published by the World Bank, which may not represent the actual resources devoted to Veterinary Services or to emergency preparedness and response. Shorter time periods from the detection of FMD to the elimination/vaccination of the last case (depending on the control strategy) in the study unit were also found to increase the likelihood of rapid recovery times after the suspension of FMD-free status. In other words, the shorter the time from detection to elimination/vaccination of the last FMD case, the shorter the time to recover FMD-free status. This demonstrates the critical importance of the capacity of Veterinary Services during the onset of the emergency, to detect and diagnose FMD, including their ability to track and trace cases both backwards and forwards, and operational efficiency and effectiveness in implementing controls on infected places. Such operations will likely reduce the scale and duration of outbreaks. Nevertheless, after elimination/vaccination of the last FMD case, the country or zone must still provide evidence of the absence of FMD, in accordance with the relevant provisions of the Terrestrial Code.

An increase in the percentage of the livestock population at risk in the study unit during the outbreak contributed to a delay in recovery time, again re-affirming the importance of controlling the size of outbreaks. This variable may have been affected by the time taken to implement control measures in the study unit (no evidence of statistical significance). Experiences of previous outbreaks in Chinese Taipei and the UK (66) provide evidence that a delay in the implementation of movement bans and shutting down of markets contributes to an increase in the size of the epidemic, which suggests that FMD spread occurred through the movement of animals in the subclinical stage of infection.

There was also evidence that the study units which shared borders with FMD-infected countries or zones were less likely to recover their FMD-free status rapidly. This is an important finding for Members to consider. They may consider national strategies that implement targeted or heightened surveillance in these border areas aimed at the early detection of FMDv introduction, as well as stricter prevention strategies and controls in the movement of animals and animal products to and from these areas, potentially (but not necessarily) within a zoning approach. The finding emphasizes the importance of regional collaboration in transboundary FMD risk management, both in preventing outbreaks and also during control operations during outbreaks.

Interestingly, it was observed that the study units that had their FMD-free status suspended within 12 months after recognition or recovery, were more likely to take a longer time to recover from a subsequent outbreak. This finding illustrates the vulnerability of countries and zones in the period after FMD recognition/recovery, and the need for follow-up work to be done to maintain the FMD-free status. This may suggest a need to prioritize resources and activities and maintain vigilance against FMD, particularly during the first year of FMD-free status, for successful or continuous maintenance of that status. This is also a relevant consideration for WOAH to put a particular emphasis in following up countries or zones during the first year after attaining FMD-free status.

The availability of public veterinarians, measured as the number of official veterinarians per 100,000 livestock, was also linked to the time taken to recover FMD-free status in the study population. If increasing the number of official veterinarians is not possible, an effective strategy might be to train and allocate more veterinarians or veterinary para-professionals in areas with a higher density of livestock or higher risk of outbreaks (e.g., border areas). In fact, through modeling exercise, it has been shown that the success of the outbreak control was impacted by the number of staff available for surveillance activities in the early phase of the emergency (67).

In terms of the impact of control strategies implemented during the outbreak(s), the application of stamping-out (only) led to shorter recovery times when compared to stamping-out with emergency vaccination to live. There was no statistically significant difference in the time taken to recover between the application of stamping-out (only) and stamping-out with emergency vaccination and remove policy. Many studies have investigated the potential impact of emergency vaccination and retain policy in FMD-free areas without vaccination. Based on the experience of FMD outbreaks in the Netherlands in 2001, Backer et al. (38) suggest that vaccination and retain policy can be a viable alternative to stamping-out, even in situations where resources are scarce. The authors suggest targeting densely populated areas for vaccination. While mentioning the economic and ethical implications of stamping-out and emergency vaccination and remove policies, Parida (68) points out that the success of emergency vaccination and retain policy is highly dependent on good traceability systems and record-keeping. Other authors argue that implementation of a vaccination and retain policy should be avoided, based on the assumption that cattle persistently infected animals could act as a disease reservoir (35). However, evidence indicates that transmission from persistently infected animals in the field is rare (69–74). Other studies have also investigated vaccination strategies and their impact on trade, and suggest that the costs of implementing emergency vaccination and retain policy lowered the overall costs of controlling the outbreak (in comparison to using stamping-out and emergency vaccination and remove policy), but that these costs were nowhere near close to the losses in trade (7, 36). For this reason, Members with significant export markets may decide that emergency vaccination and retain policy is not the most economic strategy. In addition, Paton et al. (39) reviewed the use of non-structural protein tests in substantiating freedom from disease and suggested that, while a vaccination and retain policy is feasible, it may involve greater financial costs due to the components of the surveillance system needed to demonstrate freedom. Another important factor to consider when planning control strategies is the psychological impact that these policies can have on producers and the major opposition by the citizens to these kind of interventions, as suggested by Davies (25) in his description of the 2001 UK epidemic.

The analysis could not find any association between the existence of a PPP related to FMD activities and the time to recover the FMD-free status. Nevertheless, the authors noted the importance of PPPs in the maintenance of animal health status and disease control in Members through increasing awareness and incentivising risk management, and Members should therefore be encouraged to provide such data when applying for official recognition or recovery of FMD-free status. In addition, the authors recommend WOAH to develop a harmonized methodology to record this type of data or develop indicators to evaluate the impact of this kind of collaboration in applicant Members in the future.

Similarly, the analysis could not find any associations between having conducted simulation exercises or simulation modeling studies prior to the suspension and the time to recover the FMD-free status. Regardless, the authors considered important that Members should be encouraged to conduct simulation exercises on a regular basis to test and increase awareness and capacity in their emergency response to control FMD outbreaks more effectively and report updates on this topic. A study by Westergaard (75) summarizes all the components needed to manage and conduct simulation exercises for highly infectious diseases and more recently WOAH developed guidelines for simulation exercises that could be used by its Members (76). With regard simulation modeling studies, their use and application has significantly increased in the past decades in developed countries that have lost their FMD-free status, such as the UK, Japan, the Netherlands and South Korea (14, 77–84). It is important to note that the studies mentioned above were conducted during or after the outbreaks. Nevertheless, simulation modeling is a useful tool to explore management strategies to control outbreaks and facilitate policy making.

In assessing the different variables involved in the time taken to recover FMD-free status, the duration of the period from suspension of FMD-free status to submission of the application for the recovery of the free status was measured as the outcome. The reason for selecting this period as the outcome was to avoid incorporating the time taken by WOAH procedures in the evaluation of the recovery application. The current outputs might have suffered from misclassification bias due to uncertainty in the categorization of some variables. This misclassification may occur as a result of differences in the production system and FMD epidemiological situation between study units during the study period. Misclassification bias has also been discussed by McLaws and Ribble (66) in their review of outbreaks in non-endemic countries. There was no evidence of non-independence (clustering) in the study subjects; however, caution should be applied because there were many study units that suffered more than one FMD-free status suspension. The assumption is that study units of a Member, shared some similarities because some variables are measured at the country level and not at the zone level, and that these should be taken into account in the analysis. Perhaps other statistical methods could be explored in the future. One possible justification for the lack of evidence of clustering could be that FMD-free status suspensions in the majority of the study units were temporally far apart during the study period, and thus their epidemiological situations could have changed. In a similar way, differences in the nature of outbreaks, such as those species affected and the magnitude of FMD spread, could also have influenced the choice of control strategies adopted in the study units and their effectiveness.

### Limitations

The following limitations of the analysis should also be noted. The FMD Chapter in the Terrestrial Code (2002 edition, and editions since 2015) indicates a 24-month deadline to apply for recovery after the suspension of FMD-free status; this was not included in other editions of the Terrestrial Code. Therefore, some study units had a longer period from suspension to recovery (up to 5 years). To avoid those study subjects with prolonged periods between the suspension of their FMD-free status and their application for recovery affecting the analysis, a period of 36 months was chosen as a threshold for the inclusion of the study units. This threshold was also chosen to include a larger proportion of study units, since 90% fall within this range. The current study does not have a large sample size (*n* = 45), and this could affect the power to detect associations between variables and the time taken to recover FMD-free status.

A zonal approach to recover the FMD-free status in only a part of the initially recognized country or zone had been shown to be a reasonable strategic approach to consider for many Members—especially in South America. Whilst the zonal approach was not included as part of this study, depending on the prevailing epidemiological situation, it could be considered to gradually recover the FMD-free status of a country or zone. One important variable that was not considered in this analysis was the effect of the season on the time taken to recover FMD-free status. In a study to evaluate factors affecting the time taken to eliminate porcine epidemic diarrhea virus (PEDv) in Canada, the authors found that PEDv was eliminated faster in the spring, summer and fall than in winter (85). The reason why season was not included in this analysis was due to the variability of climates in the Members that formed part of the study sample, which did not allow the authors to make a sound comparison. Other variables were removed from the study because of the large number of missing values. Dohoo (86) and Pedersen et al. (87) have described methods to deal with missing values during the analysis. The same authors have explored the use of multiple imputation to account for missing values in the data (87, 88), although this method has been questioned by other authors because biased estimates have been noted in the association between predictors of interest and outcome (89, 90). Owing to the significance of the outputs of this report, methods to deal with missing values were not implemented. Nonetheless, it would be interesting to use the multiple imputation approach to conduct future analyses and assess the behavior of the models. Finally, the outputs generated by this analysis should be interpreted cautiously because of continuous improvements in the performance of surveillance and early warning systems, and increased capacity building in Members' emergency management capacity since the last suspension of their FMD-free status.

## Conclusions

This is the first project that attempts to describe the suspensions and recoveries FMD-free status in WOAH Members and to evaluate the effects of different risk factors on the time taken to recover official FMD-free status. The analysis identified important areas to be strengthened for better preparedness and contingency planning against a potential incursion of FMDv into Members' territories. Nevertheless, the authors emphasize that the findings should be considered carefully as the study made a retrospective analysis and many of the areas discussed in the sections above are likely to have improved in the years after the suspension and recovery of FMD-free status. The study also emphasizes the challenges encountered by the authors when collecting, cleaning, and analyzing the data. For this reason, the authors recommend WOAH to develop better data management strategies so that similar studies can be more readily repeated in the future and make more efficient use of the data available and produce more robust findings.

## Data availability statement

The original contributions presented in the study are included in the article/Supplementary material, further inquiries can be directed to the corresponding authors.

## Author contributions

This study was conceived by WOAH. The data collection, entry, and cleaning were done by AC. AC, PT, and MS-V contributed to the statistical analyses. All authors contributed to the study design, wrote the report, read, and approved the final version for publication.

## Funding

This project was supported by Agriculture and Agri-Food Canada (AAFC), through a funding contribution (Letter Ref. AD/ARB/2018/483) to the World Organisation for Animal Health (WOAH).

## Conflict of interest

The authors declare that the research was conducted in the absence of any commercial or financial relationships that could be construed as a potential conflict of interest. The reviewer ME declared a past co-authorship with one of the author MS-V to the handling editor.

## Publisher's note

All claims expressed in this article are solely those of the authors and do not necessarily represent those of their affiliated organizations, or those of the publisher, the editors and the reviewers. Any product that may be evaluated in this article, or claim that may be made by its manufacturer, is not guaranteed or endorsed by the publisher.

## Footnotes

^1^A part of the country defined by the Veterinary Authority, containing an animal population or subpopulation with respect to its FMD status.

^2^Handistatus II can be accessed at: https://www.woah.org/en/what-we-do/animal-health-and-welfare/disease-data-collection/data-before-2005/.

^3^WAHIS Interface can be accessed at: https://wahis.woah.org/#/home.

^4^FAOSTAT can be accessed at: http://www.fao.org/faostat/en/#data.

^5^DataBank can be accessed at: https://databank.worldbank.org/reports.aspx?source=world-development-indicators.
