# Supplementary material for: Analysis of suspensions and recoveries of official foot and mouth disease free status of WOAH Members between 1996 and 2020
Source: Front Vet Sci. 2022 Oct 28;9:1013768. doi: 10.3389/fvets.2022.1013768 (PMC9650142; doi:10.3389/fvets.2022.1013768)
Supplement: Supplementary file 1 [file Data_Sheet_1.docx]

Table A1 – Waiting periods before the Member can apply for recovery of FMD-free status, based on the strategy(s) implemented in the country or zone according to the different editions of the FMD Chapter of the *Terrestrial Code*

| **Official FMD-free status** | **Strategy(s) implemented to control the FMD outbreak** | **Waiting period (in months) in the FMD Chapter of the *Terrestrial Code*** | | | | |
| --- | --- | --- | --- | --- | --- | --- |
|  |  | **1996-2001** | **2002** | **2003-2004** | **2005-2014** | **2015-2022** |
| **Without vaccination** | Stamping-out | 3 | 3 | 3 | 3 | 3 |
|  | Stamping-out + emergency vaccination | 3 |  |  |  |  |
|  | Stamping-out + emergency vaccination to kill |  | 3 | 3 | 3 | 3 |
|  | Stamping-out + emergency vaccination to live |  | 6 | 6 | 6 | 6 |
|  | No stamping-out |  |  |  | 12 | 12 |
|  | Gain FMD-free status with vaccination^1^ |  |  |  |  | 6 |
| **With vaccination** | Stamping-out | 12 | 12 | 12^3^ |  |  |
|  | No stamping-out | 24 |  |  |  |  |
|  | Emergency vaccination |  |  | 18 | 18 | 12 |
|  | Stamping-out + emergency vaccination |  | 6 | 6 | 6 | 6 |
|  | No stamping-out and no emergency vaccination |  |  |  |  | 24 |
| **Both^2^** | Containment zone |  |  |  | 1^4^ | 1 |

^*^ Blank cells mean that Articles relative to those control strategies were not included in that edition of the *Terrestrial Code*

^1^ Where stamping-out has been applied and continues vaccination has been adopted

^2^ Applies to FMD-free status with and without vaccination

^3^ Included in the FMD Chapter of the *Terrestrial Code* only in 2003

^4^ Included in the FMD Chapter of the *Terrestrial Code* since 2008

Table A2 – Summary statistics for categorical variables

| Group  *Variable* | Scale of measurement | Number of study units (%) |
| --- | --- | --- |
| Agricultural characteristics of the study unit |  |  |
| *Epidemiological unit* | Farm | 31 (69%) |
|  | Village | 4 (9%) |
|  | Other | 10 (22%) |
| *Shared borders with neighbouring FMD-infected countries or zones* | Yes | 35 (78%) |
|  | No | 10 (22%) |
| Characteristics of the FMD outbreak |  |  |
| *FMDv serotype* | A | 1 (1%) |
|  | O | 29 (64%) |
|  | SAT 1 | 3 (7%) |
|  | SAT 2 | 7 (16%) |
|  | ASIA 1 | 2 (5%) |
|  | Multiple | 3 (7%) |
| *Species in which FMD was first detected* | Bovines | 31 (68%) |
|  | Swine | 7 (16%) |
|  | Small Ruminants | 3 (7%) |
|  | Wild | 1 (2%) |
|  | Multiple | 3 (7%) |
| *Species affected during the outbreak* | Bovines | 22 (49%) |
|  | Swine | 4 (9%) |
|  | Small ruminants | 1 (2%) |
|  | Multiple | 18 (40%) |
| Emergency response and preparedness of the study unit |  |  |
| *Income level* | High | 14 (31%) |
|  | Upper middle | 23 (51%) |
|  | Lower middle | 8 (18%) |
| *Control strategy used during the outbreak* | Stamping-out | 18 (40%) |
|  | Emergency vaccination to-live | 19 (42%) |
|  | Emergency vaccination to-kill | 8 (18%) |
| *Conduction of simulation exercises prior suspension* | Yes | 4 (8%) |
|  | No | 41 (91%) |
| *Conduction of simulation modelling studies prior suspension* | Yes | 8 (18%) |
|  | No | 37 (82%) |
| *Existence of a public private partnership^1^* | Yes | 11 (24%) |
|  | No | 30 (67%) |

^1^ In 4 study units, a PPP was in place but the starting date of the PPP could not be determined

Table A3 – Summary statistics for quantitative variables

| **Group**  *Variable* | **25^th^ Percentile** | **Median** | **75^th^ Percentile** |
| --- | --- | --- | --- |
| **Agricultural characteristics of the study unit** |  |  |  |
| *Livestock density^1^* | 0.08 | 0.38 | 0.8 |
| **Characteristics of the FMD outbreak** |  |  |  |
| *Percentage of at-risk livestock during the outbreak ^2^* | 1 | 2 | 9 |
| **Emergency response and preparedness of the study unit** |  |  |  |
| *Capacity of official veterinary services^3^* | 2 | 3 | 6.5 |
| *Time (months) since FMD freedom^4^* | 15 | 46 | 58 |
| *Time (days) taken to implement control measures after FMD detection* | 1 | 2 | 6 |
| *Time (weeks) between first detection of FMD and culling or vaccination of the last case* | 4 | 10 | 28.5 |
| *Time (years) since adoption of FMD legislation or latest revision prior to suspension of FMD-free status* | 14 | 23.5 | 32.5 |

^1^ Number of livestock per km^2^ of agricultural land

^2^ Percentage of confirmed FMD cases, and animals culled (if only stamping-out was applied) or proportion of vaccinated animals (if only emergency vaccination was applied) or animals culled and vaccinated (if stamping-out and emergency vaccination were applied) in relation to the total livestock population

^3^ number of official veterinarians per 100,000 livestock in the study unit

^4^ Refers to the time elapsed since the date of initial recognition for countries or zones that had only one suspension, or since the date of last suspension for countries or zones with more than one suspension
